# Effects of BA.1/BA.2 subvariant, vaccination and prior infection on infectiousness of SARS-CoV-2 omicron infections

**DOI:** 10.1093/jtm/taac068

**Published:** 2022-05-27

**Authors:** Suelen H Qassim, Hiam Chemaitelly, Houssein H Ayoub, Sawsan AlMukdad, Patrick Tang, Mohammad R Hasan, Hadi M Yassine, Hebah A Al-Khatib, Maria K Smatti, Hanan F Abdul-Rahim, Gheyath K Nasrallah, Mohamed Ghaith Al-Kuwari, Abdullatif Al-Khal, Peter Coyle, Anvar Hassan Kaleeckal, Riyazuddin Mohammad Shaik, Ali Nizar Latif, Einas Al-Kuwari, Andrew Jeremijenko, Adeel A Butt, Roberto Bertollini, Hamad Eid Al-Romaihi, Mohamed H Al-Thani, Laith J Abu-Raddad

**Affiliations:** Infectious Disease Epidemiology Group, Weill Cornell Medicine-Qatar, Cornell University, Doha, Qatar; World Health Organization Collaborating Centre for Disease Epidemiology Analytics on HIV/AIDS, Sexually Transmitted Infections, and Viral Hepatitis, Weill Cornell Medicine–Qatar, Cornell University, Qatar Foundation – Education City, Doha, Qatar; Department of Population Health Sciences, Weill Cornell Medicine, Cornell University, New York, NY, USA; Infectious Disease Epidemiology Group, Weill Cornell Medicine-Qatar, Cornell University, Doha, Qatar; World Health Organization Collaborating Centre for Disease Epidemiology Analytics on HIV/AIDS, Sexually Transmitted Infections, and Viral Hepatitis, Weill Cornell Medicine–Qatar, Cornell University, Qatar Foundation – Education City, Doha, Qatar; Department of Population Health Sciences, Weill Cornell Medicine, Cornell University, New York, NY, USA; Mathematics Program, Department of Mathematics, Statistics, and Physics, College of Arts and Sciences, Qatar University, Doha, Qatar; Infectious Disease Epidemiology Group, Weill Cornell Medicine-Qatar, Cornell University, Doha, Qatar; World Health Organization Collaborating Centre for Disease Epidemiology Analytics on HIV/AIDS, Sexually Transmitted Infections, and Viral Hepatitis, Weill Cornell Medicine–Qatar, Cornell University, Qatar Foundation – Education City, Doha, Qatar; Department of Pathology, Sidra Medicine, Doha, Qatar; Department of Pathology, Sidra Medicine, Doha, Qatar; Biomedical Research Center, Member of QU Health, Qatar University, Doha, Qatar; Department of Biomedical Science, College of Health Sciences, Member of QU Health, Qatar University, Doha, Qatar; Biomedical Research Center, Member of QU Health, Qatar University, Doha, Qatar; Department of Biomedical Science, College of Health Sciences, Member of QU Health, Qatar University, Doha, Qatar; Biomedical Research Center, Member of QU Health, Qatar University, Doha, Qatar; Department of Biomedical Science, College of Health Sciences, Member of QU Health, Qatar University, Doha, Qatar; Department of Public Health, College of Health Sciences, QU Health, Qatar University, Doha, Qatar; Biomedical Research Center, Member of QU Health, Qatar University, Doha, Qatar; Department of Biomedical Science, College of Health Sciences, Member of QU Health, Qatar University, Doha, Qatar; Primary Health Care Corporation, Doha, Qatar; Hamad Medical Corporation, Doha, Qatar; Biomedical Research Center, Member of QU Health, Qatar University, Doha, Qatar; Hamad Medical Corporation, Doha, Qatar; Wellcome-Wolfson Institute for Experimental Medicine, Queens University, Belfast, UK; Hamad Medical Corporation, Doha, Qatar; Hamad Medical Corporation, Doha, Qatar; Hamad Medical Corporation, Doha, Qatar; Hamad Medical Corporation, Doha, Qatar; Hamad Medical Corporation, Doha, Qatar; Department of Population Health Sciences, Weill Cornell Medicine, Cornell University, New York, NY, USA; Hamad Medical Corporation, Doha, Qatar; Department of Medicine, Weill Cornell Medicine, Cornell University, New York, NY, USA; Ministry of Public Health, Doha, Qatar; Ministry of Public Health, Doha, Qatar; Ministry of Public Health, Doha, Qatar; Infectious Disease Epidemiology Group, Weill Cornell Medicine-Qatar, Cornell University, Doha, Qatar; World Health Organization Collaborating Centre for Disease Epidemiology Analytics on HIV/AIDS, Sexually Transmitted Infections, and Viral Hepatitis, Weill Cornell Medicine–Qatar, Cornell University, Qatar Foundation – Education City, Doha, Qatar; Department of Population Health Sciences, Weill Cornell Medicine, Cornell University, New York, NY, USA; Department of Public Health, College of Health Sciences, QU Health, Qatar University, Doha, Qatar

**Keywords:** COVID-19, Omicron, subvariant, sub-lineage, vaccine, breakthrough infection, immunity, epidemiology

##  

Qatar experienced a large severe acute respiratory syndrome coronavirus 2 (SARS-CoV-2) Omicron (B.1.1.529) wave that started on 19 December 2021 and peaked in mid-January, 2022.^1^ We investigated effects of Omicron subvariant (BA.1 and BA.2), previous vaccination and prior infection on infectiousness of Omicron infections, between 23 December 2021 and 20 February 2022. Incidence was initially dominated by BA.1, but within a few days, BA.2 predominated (Supplementary Figure S1 and Supplementary Section S1, Supplementary Appendix).

The quantitative reverse transcription polymerase chain reaction (RT-qPCR) cycle threshold (Ct) value of a SARS-CoV-2 infection represents the inverse of viral load and is correlated with culturable virus; thus, it can be used as a proxy for SARS-CoV-2 infectiousness.^2^^,^^3^ Accordingly, a low Ct value implies high infectiousness.

**Table 1:** Associations with RT-qPCR Ct value among 156 202 individuals with SARS-CoV-2 Omicron infection between 23 December 2021 and 20 February 2022

Characteristics	RT-qPCR Ct value	Univariable analysis		F-test^a^	Multivariable analysis^b^	
	Mean (SD)	β coefficient [95% CI]	*P*-value	*P*-value	β coefficient [95% CI]	*P*-value
Age group in years				<0.001		
10–19^c^	24.56 (6.13)	Ref.			Ref.	
<10	27.48 (5.85)	2.92 [2.77, 3.07]	<0.001		2.99 [2.84, 3.13]	<0.001
20–29	24.29 (6.11)	−0.26 [−0.39, −0.14]	<0.001		−0.03 [−0.15, 0.08]	0.568
30–39	23.83 (6.07)	−0.73 [−0.84, −0.61]	<0.001		−0.30 [−0.41, −0.19]	<0.001
40–49	23.82 (6.12)	−0.73 [−0.86, −0.61]	<0.001		−0.38 [−0.50, −0.25]	<0.001
50–59	23.51 (6.18)	−1.05 [−1.20, −0.91]	<0.001		−0.79 [−0.93, −0.65]	<0.001
60–69	23.52 (6.19)	−1.04 [−1.24, −0.85]	<0.001		−1.03 [−1.21, −0.84]	<0.001
70–79	22.84 (6.06)	−1.72 [−2.07, −1.38]	<0.001		−1.67 [−1.99, −1.35]	<0.001
80+	22.30 (5.87)	−2.25 [−2.78, −1.73]	<0.001		−2.09 [−2.57, −1.61]	<0.001
Sex				<0.001		
Female	24.11 (6.18)	Ref.			Ref.	
Male	24.28 (6.16)	0.17 [0.10, 0.23]	<0.001		0.24 [0.18, 0.30]	
Nationality^d^				<0.001		
Qatari	24.56 (6.08)	Ref.			Ref.	
Bangladeshi	24.27 (6.48)	−0.29 [−0.48, −0.10]	0.003		0.33 [0.15, 0.51]	<0.001
Egyptian	23.37 (5.87)	−1.19 [−1.34, −1.04]	<0.001		−0.41 [−0.55, −0.27]	<0.001
Filipino	22.89 (5.88)	−1.67 [−1.78, −1.57]	<0.001		−0.96 [−1.07, −0.85]	<0.001
Indian	24.48 (6.33)	−0.09 [−0.18, 0.01]	0.072		0.08 [−0.01, 0.18]	0.083
Nepalese	25.25 (6.34)	0.69 [0.53, 0.84]	<0.001		1.06 [0.91, 1.21]	<0.001
Pakistani	24.37 (6.24)	−0.19 [−0.38, −0.00]	0.044		0.29 [0.12, 0.46]	0.001
Sri Lankan	24.26 (6.24)	−0.30 [−0.50, −0.10]	0.003		0.18 [−0.01, 0.36]	0.062
Sudanese	24.11 (5.97)	−0.46 [−0.64, −0.27]	<0.001		0.58 [0.41, 0.74]	<0.001
Other nationalities^e^	24.30 (6.14)	−0.27 [−0.36, −0.18]	<0.001		−0.07 [−0.16, 0.01]	0.088
Omicron subvariant				<0.001		
BA.1	27.11 (6.60)	Ref.			Ref.	
BA.2	23.46 (5.82)	−3.65 [−3.73, −3.58]	<0.001		−3.53 [−3.60, −3.46]	<0.001
Reason for RT-qPCR testing				<0.001		
Survey	24.20 (6.17)	Ref.			Ref.	
Clinical suspicion	22.00 (5.52)	−2.20 [−2.31, −2.09]	<0.001		−1.99 [−2.09, −1.89]	<0.001
Contact tracing	24.78 (6.24)	0.58 [0.46, 0.70]	<0.001		−0.44 [−0.56, −0.33]	<0.001
Healthcare routine testing	23.79 (6.05)	−0.41 [−0.67, −0.15]	0.002		−0.52 [−0.76, −0.28]	<0.001
Port of entry	26.62 (6.17)	2.42 [2.26, 2.58]	<0.001		1.30 [1.14, 1.45]	<0.001
Pre-travel	25.38 (6.16)	1.18 [1.08, 1.29]	<0.001		0.67 [0.57, 0.77]	<0.001
Individual request	24.31 (5.99)	0.12 [−0.03, 0.26]	0.112		−0.10 [−0.23, 0.04]	0.149
Other	23.74 (5.67)	−0.45 [−1.11, 0.20]	0.171		−0.87 [−1.48, −0.27]	0.005
RT-qPCR test study-period week				<0.001		
Week 1 (23–29 December 2021)	23.39 (5.90)	Ref.			Ref.	
Week 2 (30 December 2021–05 January 2022)	23.31 (5.90)	−0.08 [−0.18, 0.03]	0.142		0.47 [0.37, 0.57]	<0.001
Week 3 (06–12 January 2022)	24.17 (6.02)	0.78 [0.67, 0.90]	<0.001		1.43 [1.32, 1.54]	<0.001
Week 4 (13–19 January 2022)	25.88 (6.23)	2.49 [2.35, 2.62]	<0.001		2.92 [2.79, 3.05]	<0.001
Week 5 (20–26 January 2022)	27.76 (6.25)	4.37 [4.20, 4.55]	<0.001		4.70 [4.53, 4.87]	<0.001
Week 6 (27 January–02 February 2022)	28.62 (6.17)	5.23 [5.02, 5.44]	<0.001		5.10 [4.90, 5.30]	<0.001
Week 7 (03–09 February 2022)	29.29 (5.99)	5.90 [5.64, 6.16]	<0.001		5.58 [5.34, 5.83]	<0.001****
Week 8 (10–16 February 2022)	28.48 (6.13)	5.09 [4.73, 5.45]	<0.001		4.73 [4.39, 5.06]	<0.001
Week 9 (17–20 February 2022)	28.10 (6.31)	4.71 [4.10, 5.31]	<0.001		4.59 [4.02, 5.15]	<0.001
Vaccination status				<0.001		
Unvaccinated	25.38 (6.27)	Ref.			Ref.	
One dose	23.92 (6.05)	−1.46 [−1.82, −1.09]	<0.001		−0.34 [−0.67, −0.00]	0.050
Two doses						
<3 months before the RT-qPCR test	24.69 (6.25)	−0.69 [−0.93, −0.44]	<0.001		0.23 [0.00, 0.46]	0.048
3– < 6 months before the RT-qPCR test	24.07 (6.16)	−1.31 [−1.42, −1.20]	<0.001		−0.05 [−0.15, 0.06]	0.389
6– < 9 months before the RT-qPCR test	23.43 (5.96)	−1.95 [−2.02, −1.87]	<0.001		−0.48 [−0.56, −0.40]	<0.001
≥9 months before the RT-qPCR test	23.47 (5.97)	−1.91 [−2.00, −1.81]	<0.001		−0.43 [−0.53, −0.33]	<0.001
Three doses						
≤1 month before the RT-qPCR test	24.98 (6.30)	−0.39 [−0.54, −0.25]	<0.001		0.86 [0.72, 1.00]	<0.001
>1 month before the RT-qPCR test	24.21 (6.23)	−1.17 [−1.31, −1.02]	<0.001		0.28 [0.14, 0.42]	<0.001
Previous SARS-CoV-2 infection				<0.001		
Never	24.09 (6.16)	Ref.			Ref.	
<90 days before the study RT-qPCR test^f^	29.18 (5.41)	5.09 [4.58, 5.60]	<0.001		4.23 [3.77, 4.69]	<0.001
Prior infection^g^	25.22 (6.07)	1.12 [1.01, 1.23]	<0.001		1.30 [1.20, 1.39]	<0.001

CI, confidence interval; Ct, cycle threshold; RT-qPCR, real-time reverse-transcription polymerase chain reaction; Ref., reference; SARS-CoV-2, severe acute respiratory syndrome coronavirus 2; SD, standard deviation.

a The two-tailed F-test of the univariable analysis.

b RT-qPCR Ct value was adjusted for age-group, sex, nationality, Omicron subvariant, reason for RT-qPCR test, RT-qPCR test study-period week, vaccination status and prior SARS-CoV-2 infection.

c The 10–19 age group was chosen as a reference, and not the < 10-age group, because of the different manifestations of this infection in small children.

d Nationalities were chosen to represent the most populous groups on Qatar.

e These comprise 44 other nationalities in Qatar.

f An RT-qPCR-positive test that occurred < 90 days before the study RT-qPCR-positive test was included separately in the analysis, but was not considered a prior infection. This RT-qPCR-positive test and the study RT-qPCR-positive test may both reflect the same prolonged infection.

g Prior infection was defined as an RT-qPCR-positive test that occurred ≥90 days before the RT-qPCR-positive test that is included in the study.

Univariable and multivariable regression analyses were conducted to estimate the association between Ct value and each of the Omicron subvariants, mRNA vaccination (factoring dose number and time since vaccination), prior infection, reason for RT-qPCR testing, calendar week of RT-qPCR testing (to account for phases of the rapidly evolving Omicron wave) and demographic factors including sex, age and nationality (Section S2). The study was reported following STROBE guidelines. The STROBE checklist is found in Supplementary Table S4.

Supplementary Figure S2 shows the process of selecting the study population and Supplementary Table S1 describes the study population characteristics. This was a national study involving 156 202 individuals infected with Omicron who are broadly representative of Qatar’s population. To standardize Ct values and ascertain subvariant status, we analysed only RT-qPCR-confirmed infections diagnosed with TaqPath COVID-19 Combo Kit (Thermo Fisher Scientific, USA), used to process most RT-qPCR tests in Qatar.^3^

Compared with BA.1, BA.2 was associated with 3.53 fewer cycles (95% confidence interval [CI]: 3.46–3.60), signifying higher infectiousness (Table 1). Ct value decreased with time since second and third vaccinations, mirroring the established pattern of waning vaccine effectiveness.^4^ Ct values were highest for those who received their boosters in the month preceding the RT-qPCR test—0.86 cycles (95% CI: 0.72–1.00) higher than for unvaccinated persons. Ct value was 1.30 (95% CI: 1.20–1.39) cycles higher for those with a prior infection compared with those without prior infection, signifying lower infectiousness.

Ct value declined gradually with age (Table 1), perhaps reflecting slower virus clearance with aging. There were differences in Ct value by sex and nationality, but these may reflect different test-seeking behaviours for different socio-economic groups in Qatar’s diverse population. Ct value was lowest for those who were tested because of symptoms and was highest for those who were tested for travel-related purposes. Ct value was lowest during the exponential-growth phase of the Omicron wave, as a large proportion of infections were recent, and was highest after the wave peaked and was declining, as a small proportion of infections were recent. Stratified analyses for BA.1 and BA.2 showed similar findings (Supplementary Tables S2 and S3). Limitations are discussed in Section S2.

The BA.2 subvariant appears substantially more infectious than the BA.1 subvariant, consistent with findings of a household study from Denmark.^5^ This may reflect higher viral load and/or longer duration of infection, thereby explaining the rapid expansion of this subvariant in Qatar (Supplementary Figure S1). Natural immunity from previous infection and strength of vaccine immunity correlates with less infectious breakthrough infections, as observed for earlier SARS-CoV-2 variants.^3^ Symptomatic infection and older age are associated with higher infectiousness.

## Author contributions

S.H.Q. co-designed the study, performed the statistical analyses and co-wrote the first draft of the article. H.C. co-designed the study, supported the statistical analyses and co-wrote the first draft of the article. L.J.A. conceived and co-designed the study, led the statistical analyses and co-wrote the first draft of the article. P.T. and M.R.H. conducted the multiplex, RT-qPCR variant screening and viral genome sequencing. H.Y., H.A.K. and M.S. conducted viral genome sequencing. All authors contributed to data collection and acquisition, database development, discussion and interpretation of the results, and to the writing of the manuscript. All authors have read and approved the final manuscript.

## Supplementary Material

Supplementary_Appendix_taac068
