# Supplementary material for: Effects of BA.1/BA.2 subvariant, vaccination and prior infection on infectiousness of SARS-CoV-2 omicron infections
Source: J Travel Med. 2022 May 27;29(6):taac068. doi: 10.1093/jtm/taac068 (PMC9213851; doi:10.1093/jtm/taac068)
Supplement: Supplementary_Appendix_taac068 [file supplementary_appendix_taac068.docx]

**Supplementary Appendix**

**Table of contents**

[**Section S1. Laboratory methods and variant ascertainment** 2](#_Toc102400131)

[**Real-time reverse-transcription polymerase chain reaction testing** 2](#_Toc102400132)

[**Classification of infections by variant type** 2](#_Toc102400133)

[**Section S2. Study population, data sources, and study design** 5](#_Toc102400134)

[**Oversight** 7](#_Toc102400135)

[**Statistical analysis** 7](#_Toc102400136)

[**Limitations** 7](#_Toc102400137)

[**Figure S1. Proportion of BA.1 (versus BA.2) Omicron infections in the PCR-positive tests assessed using TaqPath COVID-19 Combo Kit during the study period.** 10](#_Toc102400138)

[**Figure S2. Flowchart describing the population selection process for investigating the infectiousness of SARS-CoV-2 Omicron variant infections.** 11](#_Toc102400139)

[**Table S1. Characteristics of included individuals with SARS-CoV-2 Omicron infections between December 23, 2021 and February 20, 2022.** 12](#_Toc102400140)

[**Table S2. Associations with RT-qPCR Ct value among 32,153 individuals with SARS-CoV-2 Omicron BA.1 subvariant infection.** 14](#_Toc102400141)

[**Table S3. Associations with RT-qPCR Ct value among 124,049 individuals with SARS-CoV-2 Omicron BA.2 subvariant infection.** 16](#_Toc102400142)

[**Table S4. STROBE checklist for cross-sectional studies.** 18](#_Toc102400143)

[**Supplementary references** 20](#_Toc102400144)

**Section S1. Laboratory methods** **and variant ascertainment**

## **Real-time reverse-transcription polymerase chain reaction testing**

Nasopharyngeal and/or oropharyngeal swabs were collected for polymerase chain reaction (PCR) testing and placed in Universal Transport Medium (UTM). Aliquots of UTM were: 1) extracted on KingFisher Flex (Thermo Fisher Scientific, USA), MGISP-960 (MGI, China), or ExiPrep 96 Lite (Bioneer, South Korea) followed by testing with real-time reverse-transcription PCR (RT-qPCR) using TaqPath COVID-19 Combo Kits (Thermo Fisher Scientific, USA) on an ABI 7500 FAST (Thermo Fisher Scientific, USA); 2) tested directly on the Cepheid GeneXpert system using the Xpert Xpress SARS-CoV-2 (Cepheid, USA); or 3) loaded directly into a Roche cobas 6800 system and assayed with the cobas SARS-CoV-2 Test (Roche, Switzerland). The first assay targets the viral S, N, and ORF1ab gene regions. The second targets the viral N and E-gene regions, and the third targets the ORF1ab and E-gene regions.

All PCR testing was conducted at the Hamad Medical Corporation Central Laboratory or Sidra Medicine Laboratory, following standardized protocols.

## **Classification of infections by variant type**

Surveillance for severe acute respiratory syndrome coronavirus 2 (SARS-CoV-2) variants in Qatar is mainly based on viral genome sequencing and multiplex RT-qPCR variant screening^1^ of random positive clinical samples,^2-7^ complemented by deep sequencing of wastewater samples.^4, 8^

A total of 315 random SARS-CoV-2-positive specimens collected between December 19, 2021 and January 22, 2022 were viral whole-genome sequenced on a Nanopore GridION sequencing device. Of these, 300 (95.2%) were confirmed as Omicron (B.1.1.529)^9^ infections and 15 (4.8%) as Delta (B.1.617.2)^9^ infections.^4, 10, 11^ Of 286 Omicron infections with confirmed subvariant status, 68 (23.8%) were BA.1 cases and 218 (76.2%) were BA.2 cases.

Additionally, a total of 1,315 random SARS-CoV-2-positive specimens collected between December 22, 2021 and January 1, 2022 were RT-qPCR genotyped. The RT-qPCR genotyping identified 1 B.1.617.2-like Delta case, 366 BA.1-like Omicron cases, 898 BA.2-like Omicron cases, and 50 were undetermined cases where the genotype could not be assigned.

The accuracy of the RT-qPCR genotyping was verified against either Sanger sequencing of the receptor-binding domain (RBD) of SARS-CoV-2 surface glycoprotein (S) gene, or by viral whole-genome sequencing on a Nanopore GridION sequencing device. From 147 random SARS-CoV-2-positive specimens all collected in December of 2021, RT-qPCR genotyping was able to assign a genotype in 129 samples. The agreement between RT-qPCR genotyping and sequencing was 100% for Delta (n=82), 100% for Omicron BA.1 (n=18), and 93% for Omicron BA.2 (27 of 29 were correctly assigned to BA.2 and remaining 2 specimens genotyped as BA.2 were B.1.617.2 by sequencing). Of the remaining 18 specimens: 10 failed PCR amplification and sequencing, 8 could not be assigned a genotype by RT-qPCR (4 of 8 were B.1.617.2 by sequencing, and the remaining 4 failed sequencing). All the variant RT-qPCR genotyping was conducted at the Sidra Medicine Laboratory following standardized protocols.

The large Omicron-wave exponential-growth phase in Qatar started on December 19, 2021 and peaked in mid-January, 2022.^4, 10-13^ The study duration coincided with the intense Omicron wave where Delta incidence was limited. Accordingly, any PCR-positive test during the study duration, between December 23, 2021 and February 20, 2022, was assumed to be an Omicron infection. Of note that the study duration started on December 23, 2021, and not on December 19, 2021, to minimize the occurrence of residual Delta incidence during the first few days of the Omicron wave.

Informed by the viral genome sequencing and the RT-qPCR genotyping, a SARS-CoV-2 infection with the BA.1 subvariant was proxied as an S-gene “target failure” (SGTF) case using the TaqPath COVID-19 Combo Kit (Thermo Fisher Scientific, USA^14^) that tests for the S-gene and is affected by the del69/70 mutation in the S-gene.^15^ A SARS-CoV-2 infection with the BA.2 subvariant was proxied as a non-SGTF case using this TaqPath Kit. For ascertainment of subvariant status and standardization of RT-qPCR cycle threshold values, we analyzed only the RT-qPCR-confirmed infections diagnosed with this TaqPath Kit.

# **Section S2. Study population, data sources, and study design**

The real-time reverse-transcription polymerase chain reaction (RT-qPCR) cycle threshold (Ct) value is a measure of the inverse of viral load and correlates strongly with culturable virus;^16^ thus, it can be used as a proxy for severe acute respiratory syndrome coronavirus 2 (SARS-CoV-2) infectiousness.^16-21^ We investigated several effects on the infectiousness of Omicron^9^ (B.1.1.529) variant infections in the resident population of Qatar using a cross-sectional study design. These included: subvariant (BA.1 versus BA.2), mRNA (BNT162b2^22^ and mRNA-1273^23^) vaccination status, prior infection status, reason for RT-qPCR testing, study-period week of the RT-qPCR test (to account for the evolving phase of the rapid Omicron wave), and demographic factors including sex, age, and nationality.

The study population included all individuals with an RT-qPCR-confirmed SARS-CoV-2 infection in Qatar between December 23, 2021 and February 20, 2022. Coronavirus disease 2019 (COVID-19) laboratory testing, vaccination, clinical infection, and demographic data for this population were extracted from the national, federated SARS-CoV-2 databases, which include all RT-qPCR testing, reason for RT-qPCR testing, COVID-19 vaccinations, and related demographic details since the start of the pandemic. Further description of Qatar’s national COVID-19 databases can be found in previous publications.^7, 21, 24-26^

Every SARS-CoV-2 RT-qPCR test conducted in Qatar is classified based on the reason for testing (clinical symptoms, contact tracing, surveys or random testing campaigns, individual requests, routine healthcare testing, pre-travel, at port of entry, or other). RT-qPCR testing is performed at a mass scale, with about 5% of the population tested every week.^7^ About 75% of those diagnosed over recent months were diagnosed not because of appearance of symptoms, but because of routine testing.^7^ Qatar has unusually young, diverse demographics, in that only 9% of its residents are ≥50 years of age, and 89% are expatriates from over 150 countries.^24, 27^ Nearly all individuals were vaccinated in Qatar, however, vaccinations performed elsewhere were still recorded in the health system at the port of entry upon arrival to Qatar per country requirements.

Qatar has been experiencing a large Omicron wave that started on December 19, 2021 and peaked in mid-January, 2022.^4, 10-13^ Initially, the BA.1 subvariant was dominant, but within days, the BA.2 subvariant predominated (Figure S1).

For each individual, we selected only the first positive RT-qPCR-confirmed swab during the study period. We subsequently derived a summary measure for our primary outcome, the RT-qPCR Ct value, by averaging the Ct values of the N, ORF1ab, and S (if not an S-gene “target failure” case) genes. This average Ct value was used as the dependent variable in all analyses.

Both vaccination status and prior infection status were ascertained at the time of the RT-qPCR test. Vaccination status was defined factoring the number of administered vaccine doses and months elapsed since the last vaccine dose, with one month defined as 30 days. Only vaccination with BNT162b2^22^ and mRNA-1273^23^ vaccines were considered in the analyses, as these have been the vaccines of choice in the COVID-19 immunization program in Qatar.^2, 3, 28^ Rare occurrences of mixed vaccination regimens were excluded. Prior infection was defined as an RT-qPCR-positive test that occurred ≥90 days before the study RT-qPCR-positive test.^12, 26, 29-36^ An RT-qPCR-positive test that occurred <90 days prior to the study RT-qPCR-positive test was still factored in the analysis, but was not considered a prior infection. This is because this RT-qPCR-positive test and the study RT-qPCR-positive test may both reflect the same prolonged infection.^37-39^ A small number of RT-qPCR tests had no recorded Ct value and were thus excluded from the analysis, but these constituted only 0.1% of all RT-qPCR tests. Otherwise, data on the remaining study variables were complete.

## **Oversight**

Hamad Medical Corporation and Weill Cornell Medicine-Qatar Institutional Review Boards approved this retrospective study with waiver of informed consent. The study was reported following STROBE guidelines. The STROBE checklist is found in Table S4.

## **Statistical analysis**

Frequency distributions and measures of central tendency were used to describe the study population with respect to a priori determined factors. These included Omicron infection subvariant, vaccination status (factoring dose number and months since vaccination), prior infection status, reason for RT-qPCR testing, study-period week of the RT-qPCR test, and demographic factors, namely sex, age, and nationality. Differences between BA.1 and BA.2 infections were estimated using standardized mean differences (SMDs).

Association of each of these factors with Ct value was assessed using univariable linear regression analyses. Unadjusted β coefficients, 95% confidence intervals (CIs), and the F-test of overall covariate significance were reported. Adjusted β coefficients and associated 95% CIs and p-values were estimated using multivariable linear regression analyses that included all covariates in the model.

The 95% CIs were not adjusted for multiplicity. Two-sided p-value <0.05 indicated statistical significance. Statistical analyses were conducted in STATA/SE version 16.^40^

**Limitations**

A small number of RT-qPCR tests had no available Ct value and were thus excluded from the analysis, but these constituted only 0.1% of all RT-qPCR tests. The study was implemented on documented RT-qPCR-confirmed infections, but other infections may have occurred but were never documented. It is possible that infections in those with prior infection or those vaccinated are less likely to be diagnosed, perhaps because of minimal or no symptoms. Nevertheless, RT-qPCR testing in Qatar is done at a mass scale, where a significant proportion of the population is being tested every week.^7^ The majority of infections are identified not because of symptoms, but because of routine testing for other reasons (Table S1).^7^ The date of symptom onset was not available for symptomatic cases. Therefore, an analysis factoring the duration between symptom onset and RT-qPCR test was not possible.

The study population consists of mostly working-age adults and thus the results may not necessarily be generalizable to other population groups, such as the elderly. The analyses controlled for sex, age, and nationality but it was not possible to control for other factors, such as comorbidities or socio-economic factors, as data on these factors were not available. Of note that the number of individuals with severe chronic conditions is small in Qatar’s young population.^24, 41^ The national list of vaccine prioritization included only 19,800 individuals of all age groups with serious co-morbid conditions to be prioritized in the first phase of vaccine roll-out.^7^ Factoring nationality in the analyses may have (partially) controlled socio-economic differences/occupational risk, in consideration of the association between nationality and occupation in Qatar.^24, 42-45^

BA.1 and BA.2 ascertainment was based on proxy criteria; presence or absence of SGTF using the TaqPath Kit, but this method of ascertainment is well established not only for Omicron subvariants, but also for other variants such as Alpha.^15, 35, 46^ Some Omicron infections may have been misclassified Delta infections, but this is not likely, as Delta incidence was limited during the study (Section S1).

BA.1 was introduced before BA.2 into Qatar’s population. The differences in Ct value between the two subvariant infections could be confounded by different phases for each of BA.1 and BA.2 expansions into the population. To account for this possibility, the regression analyses were repeated but with an interaction term between Omicron infection subvariant and study-period week of the RT-qPCR test. The differences in Ct value between BA.1 and BA.2 infections were maintained indicating that the different Ct values for BA.1 and BA.2 infections are not likely to be only a consequence of different phases for each of BA.1 and BA.2 expansions into the population.

Time since vaccination was associated with lower Ct value, mirroring the established pattern of waning vaccine effectiveness.^13^ Unexpectedly, however, the Ct value for those who had their second dose >6 months earlier was lower than that among unvaccinated persons. With the high vaccine coverage in Qatar (exceeding 85%), the unvaccinated group may be different in other uncontrolled attributes from the vaccinated group. For example, those unvaccinated may have chosen not to receive the vaccine because of undocumented prior infection and thus are not truly immune naïve.

# **Figure S1. Proportion of BA.1 (versus BA.2) Omicron infections in the PCR-positive tests assessed using TaqPath COVID-19 Combo Kit during the study period.**

**
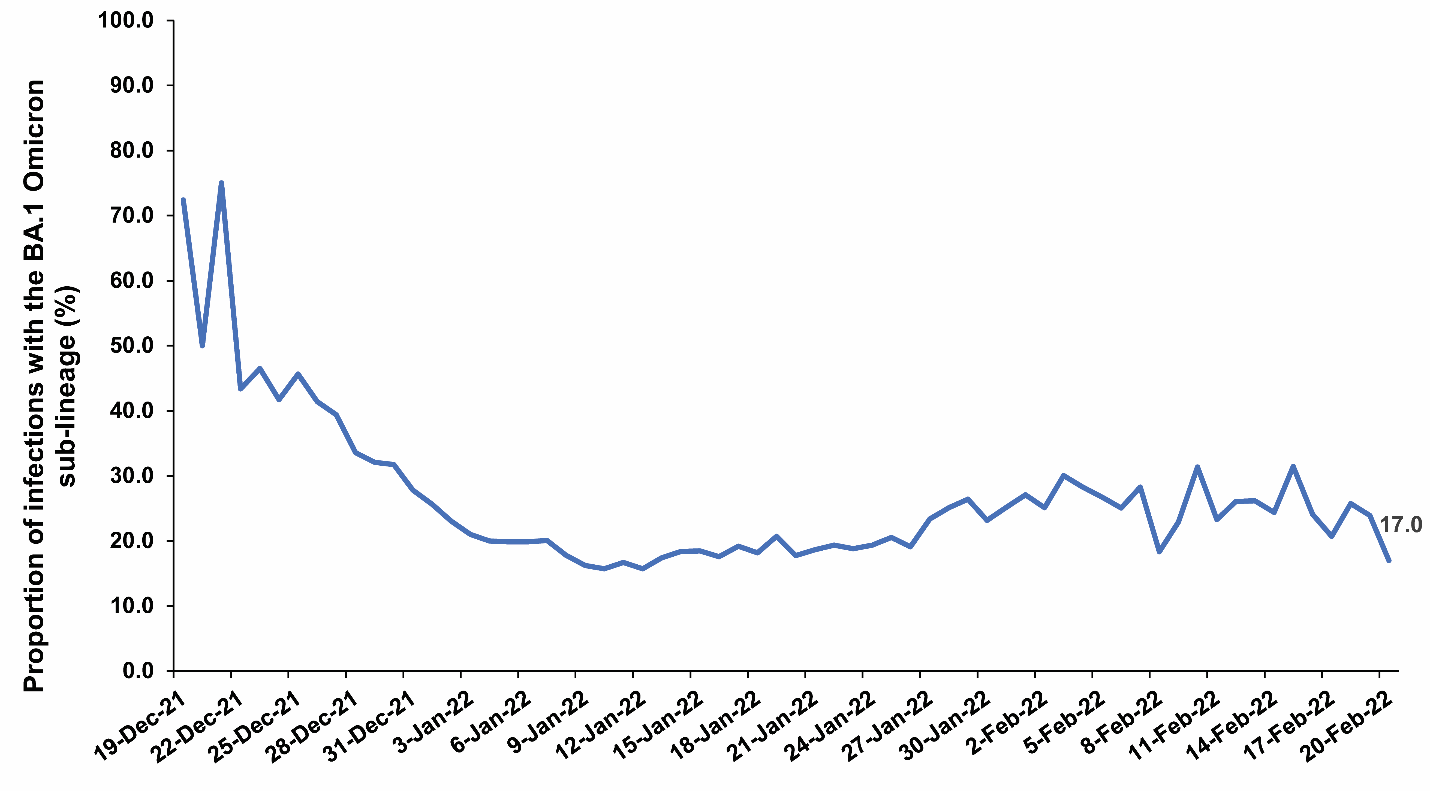
**

# **Figure S2. Flowchart describing the population selection process for investigating the infectiousness of SARS-CoV-2 Omicron variant infections.**


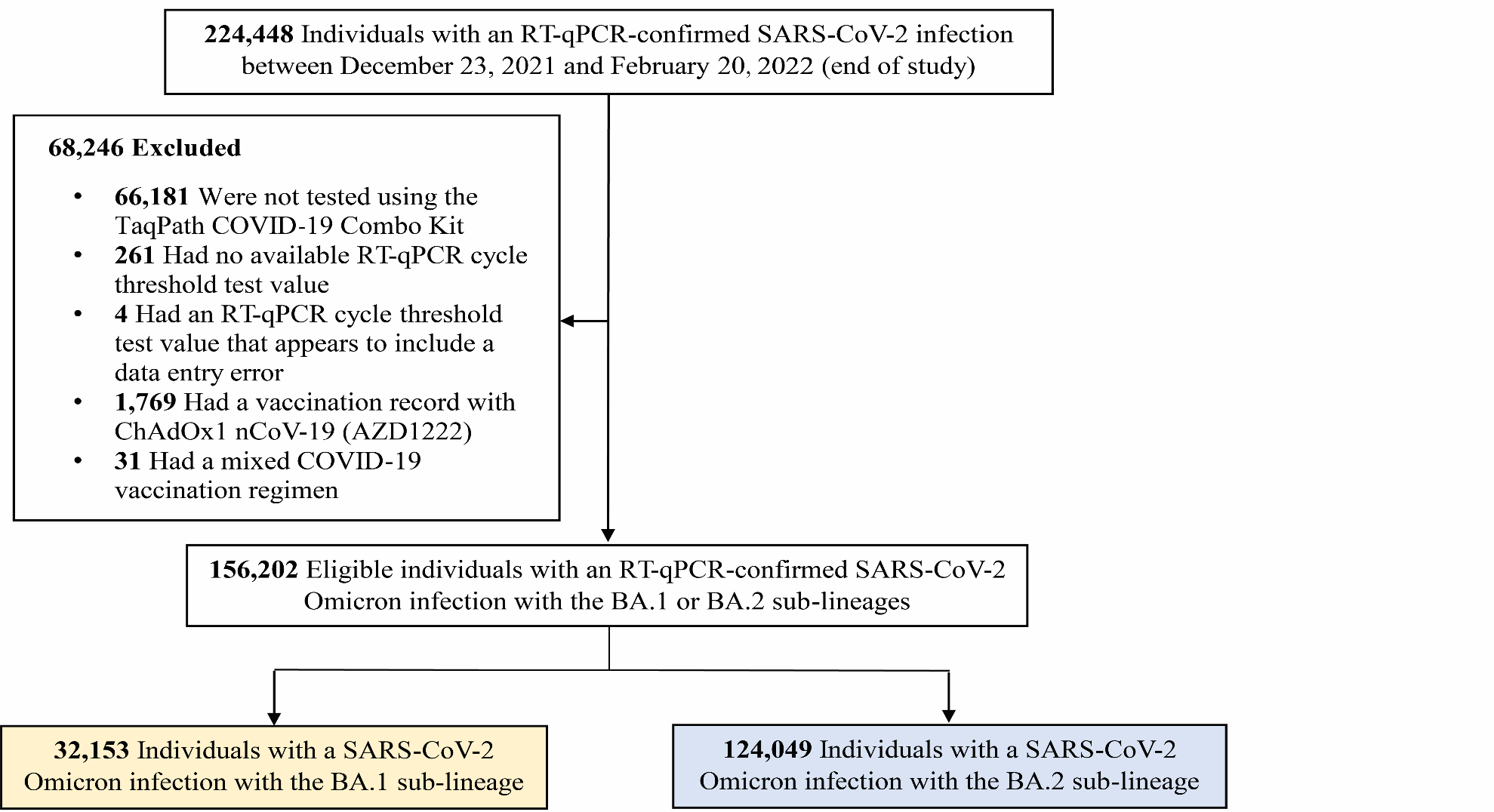


Abbreviations: COVID-19, coronavirus disease 2019; RT-qPCR, real-time reverse-transcription polymerase chain reaction; SARS-CoV-2, severe acute respiratory syndrome coronavirus 2.

# **Table S1. Characteristics of included individuals with SARS-CoV-2 Omicron infections between December 23, 2021 and February 20, 2022.**

| **Characteristics** | **Overall population**  **N (%)** | **Individuals with BA.1 infection**  **N (%)** | **Individuals with BA.2 infection**  **N (%)** | **SMD**^*^ |
| --- | --- | --- | --- | --- |
| **Total N** | 156,202 | 32,153 (20.6) | 124,049 (79.4) | - |
| **Demographic characteristics** |  |  |  |  |
| **Median age (IQR) — years** | 33 (25-42) | 32 (24-41) | 34 (26-42) | -0.09 |
| **Age group in years — no. (%)** |  |  |  |  |
| <10 | 11,797 (7.6) | 2,574 (8.0) | 9,223 (7.4) | 0.11 |
| 10-19 | 13,881 (8.9) | 3,566 (11.1) | 10,315 (8.3) |  |
| 20-29 | 31,723 (20.3) | 6,808 (21.2) | 24,915 (20.1) |  |
| 30-39 | 50,689 (32.5) | 9,969 (31.0) | 40,720 (32.8) |  |
| 40-49 | 27,452 (17.6) | 5,417 (16.8) | 22,035 (17.8) |  |
| 50-59 | 13,725 (8.8) | 2,555 (7.9) | 11,170 (9.0) |  |
| 60-69 | 5,070 (3.2) | 962 (3.0) | 4,108 (3.3) |  |
| 70-79 | 1,334 (0.9) | 225 (0.7) | 1,109 (0.9) |  |
| 80+ | 531 (0.3) | 77 (0.2) | 454 (0.4) |  |
| **Sex** |  |  |  |  |
| Female | 64,375 (41.2) | 14,585 (45.4) | 49,790 (40.1) | 0.11 |
| Male | 91,827 (58.8) | 17,568 (54.6) | 74,259 (59.9) |  |
| **Nationality**^†^ |  |  |  |  |
| Bangladeshi | 4,593 (2.9) | 684 (2.1) | 3,909 (3.2) | 0.20 |
| Egyptian | 7,614 (4.9) | 1,363 (4.2) | 6,251 (5.0) |  |
| Filipino | 19,241 (12.3) | 3,600 (11.2) | 15,641 (12.6) |  |
| Indian | 32,007 (20.5) | 5,649 (17.6) | 26,358 (21.2) |  |
| Nepalese | 7,276 (4.7) | 1,185 (3.7) | 6,091 (4.9) |  |
| Pakistani | 4,790 (3.1) | 860 (2.7) | 3,930 (3.2) |  |
| Qatari | 33,633 (21.5) | 8,218 (25.6) | 25,415 (20.5) |  |
| Sri Lankan | 4,066 (2.6) | 629 (2.0) | 3,437 (2.8) |  |
| Sudanese | 5,064 (3.2) | 1,007 (3.1) | 4,057 (3.3) |  |
| Other nationalities^‡^ | 37,918 (24.3) | 8,958 (27.9) | 28,960 (23.3) |  |
| **RT-qPCR test characteristics** |  |  |  |  |
| **Reason for RT-qPCR testing** |  |  |  |  |
| Clinical suspicion | 42,248 (27.0) | 6,800 (21.1) | 35,448 (28.6) | 0.36 |
| Contact tracing | 21,885 (14.0) | 3,619 (11.3) | 18,266 (14.7) |  |
| Healthcare routine testing | 2,377 (1.5) | 432 (1.3) | 1,945 (1.6) |  |
| Survey | 16,385 (10.5) | 3,944 (12.3) | 12,441 (10.0) |  |
| Port of entry | 7,661 (4.9) | 3,571 (11.1) | 4,090 (3.3) |  |
| Pre-travel | 53,747 (34.4) | 11,530 (35.9) | 42,217 (34.0) |  |
| Individual request | 11,567 (7.4) | 2,206 (6.9) | 9,361 (7.5) |  |
| Other | 332 (0.2) | 51 (0.2) | 281 (0.2) |  |
| **RT-qPCR test study-period week** |  |  |  |  |
| Week 1 (23-29 December, 2021) | 14,963 (9.6) | 5,419 (16.9) | 9,544 (7.7) | 0.34 |
| Week 2 (30 December, 2021-05 January, 2022) | 74,781 (47.9) | 15,836 (49.3) | 58,945 (47.5) |  |
| Week 3 (06-12 January, 2022) | 38,392 (24.6) | 5,927 (18.4) | 32,465 (26.2) |  |
| Week 4 (13-19 January, 2022) | 14,028 (9.0) | 2,223 (6.9) | 11,805 (9.5) |  |
| Week 5 (20-26 January, 2022) | 6,313 (4.0) | 956 (3.0) | 5,357 (4.3) |  |
| Week 6 (27 January-02 February, 2022) | 3,904 (2.5) | 891 (2.8) | 3,013 (2.4) |  |
| Week 7 (03-09 February, 2022) | 2,291 (1.5) | 554 (1.7) | 1,737 (1.4) |  |
| Week 8 (10-16 February, 2022) | 1,145 (0.7) | 272 (0.8) | 873 (0.7) |  |
| Week 9 (17-20 February, 2022) | 385 (0.2) | 75 (0.2) | 310 (0.2) |  |
| **Vaccine and natural immunity** |  |  |  |  |
| **Vaccination status** |  |  |  |  |
| Unvaccinated | 45,136 (28.9) | 9,801 (30.5) | 35,335 (28.5) | 0.08 |
| One dose | 1,082 (0.7) | 196 (0.6) | 886 (0.7) |  |
| Two doses |  |  |  |  |
| <3 months before the RT-qPCR test | 2,493 (1.6) | 604 (1.9) | 1,889 (1.5) |  |
| 3-<6 months before the RT-qPCR test | 17,348 (11.1) | 3,259 (10.1) | 14,089 (11.4) |  |
| 6-<9 months before the RT-qPCR test | 50,678 (32.4) | 9,946 (30.9) | 40,732 (32.8) |  |
| ≥9 months before the RT-qPCR test | 23,402 (15.0) | 5,133 (16.0) | 18,269 (14.7) |  |
| Three doses |  |  |  |  |
| ≤1 month before the RT-qPCR test | 7,890 (5.1) | 1,485 (4.6) | 6,405 (5.2) |  |
| >1 month before the RT-qPCR test | 8,173 (5.2) | 1,729 (5.4) | 6,444 (5.2) |  |
| **Previous SARS-CoV-2 infection** |  |  |  |  |
| Never | 141,839 (90.8) | 29,051 (90.4) | 112,788 (90.9) | 0.08 |
| <90 days before the study RT-qPCR test^§^ | 560 (0.4) | 255 (0.8) | 305 (0.2) |  |
| Prior infection^¶^ | 13,803 (8.8) | 2,847 (8.9) | 10,956 (8.8) |  |

Abbreviations: IQR, interquartile range; RT-qPCR, real-time reverse-transcription polymerase chain reaction; SARS-CoV-2, severe acute respiratory syndrome coronavirus 2; SMD, standardized mean difference.

^*^SMD is the difference in the mean of a covariate between groups divided by the pooled standard deviation. An SMD <0.1 indicates minimal differences between groups.

^†^Nationalities were chosen to represent the most populous groups in Qatar.

^‡^These comprise 44 other nationalities in Qatar.

^§^An RT-qPCR-positive test that occurred <90 days before the study RT-qPCR-positive test was included separately in the analysis, but was not considered a prior infection. This RT-qPCR-positive test and the study RT-qPCR-positive test may both reflect the same prolonged infection.

^¶^Prior infection was defined as an RT-qPCR-positive test that occurred ≥90 days before the RT-qPCR-positive test that is included in the study.

# **Table S2. Associations with RT-qPCR Ct value among 32,153 individuals with SARS-CoV-2 Omicron BA.1 subvariant infection.**

| **Characteristics** | **RT-qPCR Ct value** | **Univariable analysis** | | **F-test**^*^ | **Multivariable analysis^†^** | |
| --- | --- | --- | --- | --- | --- | --- |
|  | **Mean (SD)** | **β coefficient [95% CI]** | **p-value** | **p-value** | **β coefficient [95% CI]** | **p-value** |
| **Age group in years** |  |  |  | <0.001 |  |  |
| 10-19^‡^ | 26.96 (6.40) | Ref. |  |  | Ref. |  |
| <10 | 30.17 (5.51) | 3.21 [2.88, 3.54] | <0.001 |  | 3.09 [2.77, 3.42] | <0.001 |
| 20-29 | 27.20 (6.52) | 0.24 [-0.03, 0.50] | 0.082 |  | 0.09 [-0.16, 0.35] | 0.469 |
| 30-39 | 26.76 (6.69) | -0.20 [-0.45, 0.05] | 0.113 |  | -0.23 [-0.48, 0.02] | 0.069 |
| 40-49 | 26.69 (6.66) | -0.27 [-0.55, 0.01] | 0.055 |  | -0.37 [-0.64, -0.10] | 0.008 |
| 50-59 | 26.69 (6.76) | -0.27 [-0.61, 0.06] | 0.105 |  | -0.56 [-0.89, -0.24] | 0.001 |
| 60-69 | 26.59 (6.68) | -0.37 [-0.83, 0.10] | 0.123 |  | -0.93 [-1.37, -0.49] | <0.001 |
| 70-79 | 25.69 (6.95) | -1.27 [-2.15, -0.39] | 0.005 |  | -1.74 [-2.56, -0.92] | <0.001 |
| 80+ | 25.14 (7.10) | -1.82 [-3.30, -0.35] | 0.015 |  | -2.41 [-3.77, -1.04] | 0.001 |
| **Sex** |  |  |  | <0.001 |  |  |
| Female | 26.67 (6.57) | Ref. |  |  | Ref. |  |
| Male | 27.48 (6.60) | 0.81 [0.67, 0.96] | <0.001 |  | 0.31 [0.16, 0.45] | <0.001 |
| **Nationality**^§^ |  |  |  | <0.001 |  |  |
| Qatari | 26.89 (6.34) | Ref. |  |  | Ref. |  |
| Bangladeshi | 28.95 (6.87) | 2.06 [1.55, 2.57] | <0.001 |  | 1.49 [1.01, 1.98] | <0.001 |
| Egyptian | 26.22 (6.45) | -0.66 [-1.04, -0.29] | 0.001 |  | -0.49 [-0.84, -0.14] | 0.006 |
| Filipino | 25.21 (6.69) | -1.67 [-1.93, -1.42] | <0.001 |  | -1.22 [-1.47, -0.96] | <0.001 |
| Indian | 28.08 (6.76) | 1.20 [0.98, 1.42] | <0.001 |  | 0.47 [0.24, 0.69] | <0.001 |
| Nepalese | 29.42 (6.55) | 2.54 [2.14, 2.93] | <0.001 |  | 1.88 [1.49, 2.26] | <0.001 |
| Pakistani | 27.60 (6.54) | 0.72 [0.26, 1.18] | 0.002 |  | 0.00 [-0.43, 0.43] | 0.999 |
| Sri Lankan | 28.38 (6.76) | 1.49 [0.96, 2.02] | <0.001 |  | 0.92 [0.42, 1.42] | <0.001 |
| Sudanese | 26.85 (6.47) | -0.03 [-0.46, 0.40] | 0.890 |  | 0.38 [-0.02, 0.78] | 0.065 |
| Other nationalities^¶^ | 27.05 (6.47) | 0.17 [-0.03, 0.36] | 0.092 |  | -0.07 [-0.26, 0.12] | 0.466 |
| **Reason for RT-qPCR testing** |  |  |  | <0.001 |  |  |
| Survey | 26.69 (6.53) | Ref. |  |  | Ref. |  |
| Clinical suspicion | 24.43 (6.43) | -2.26 [-2.51, -2.01] | <0.001 |  | -2.20 [-2.44, -1.96] | <0.001 |
| Contact tracing | 28.10 (6.78) | 1.42 [1.13, 1.71] | <0.001 |  | -0.03 [-0.30, 0.25] | 0.854 |
| Healthcare routine testing | 27.28 (6.53) | 0.59 [-0.05, 1.23] | 0.069 |  | 0.02 [-0.58, 0.62] | 0.943 |
| Port of entry | 27.65 (6.09) | 0.96 [0.67, 1.25] | <0.001 |  | 0.52 [0.24, 0.79] | <0.001 |
| Pre-travel | 28.31 (6.37) | 1.63 [1.39, 1.86] | <0.001 |  | 0.61 [0.38, 0.83] | <0.001 |
| Individual request | 27.37 (6.50) | 0.68 [0.34, 1.01] | <0.001 |  | 0.09 [-0.22, 0.41] | 0.569 |
| Other | 25.86 (6.93) | -0.83 [-2.61, 0.95] | 0.360 |  | -1.32 [-2.98, 0.35] | 0.121 |
| **RT-qPCR** **test study-period week** |  |  |  | <0.001 |  |  |
| Week 1 (23-29 December, 2021) | 25.81 (6.39) | Ref. |  |  | Ref. |  |
| Week 2 (30 December, 2021-05 January, 2022) | 25.96 (6.40) | 0.14 [-0.05, 0.34] | 0.148 |  | 0.17 [-0.02, 0.35] | 0.083 |
| Week 3 (06-12 January, 2022) | 27.76 (6.50) | 1.94 [1.71, 2.18] | <0.001 |  | 1.69 [1.46, 1.91] | <0.001 |
| Week 4 (13-19 January, 2022) | 30.32 (5.99) | 4.50 [4.19, 4.81] | <0.001 |  | 3.97 [3.66, 4.27] | <0.001 |
| Week 5 (20-26 January, 2022) | 32.08 (5.36) | 6.27 [5.84, 6.70] | <0.001 |  | 5.65 [5.23, 6.07] | <0.001 |
| Week 6 (27 January-02 February, 2022) | 32.32 (5.04) | 6.51 [6.06, 6.95] | <0.001 |  | 5.84 [5.40, 6.27] | <0.001 |
| Week 7 (03-09 February, 2022) | 33.11 (4.36) | 7.29 [6.74, 7.84] | <0.001 |  | 6.70 [6.16, 7.23] | <0.001 |
| Week 8 (10-16 February, 2022) | 32.04 (5.15) | 6.23 [5.46, 6.99] | <0.001 |  | 5.48 [4.74, 6.22] | <0.001 |
| Week 9 (17-20 February, 2022) | 31.29 (5.53) | 5.48 [4.04, 6.91] | <0.001 |  | 5.10 [3.72, 6.48] | <0.001 |
| **Vaccination status** |  |  |  | <0.001 |  |  |
| Unvaccinated | 28.36 (6.40) | Ref. |  |  | Ref. |  |
| One dose | 27.24 (6.63) | -1.12 [-2.04, -0.19] | 0.018 |  | -0.46 [-1.32, 0.39] | 0.288 |
| Two doses |  |  |  |  |  |  |
| <3 months before the RT-qPCR test | 27.19 (6.39) | -1.17 [-1.71, -0.63] | <0.001 |  | 0.08 [-0.42, 0.58] | 0.758 |
| 3-<6 months before the RT-qPCR test | 26.98 (6.72) | -1.38 [-1.63, -1.12] | <0.001 |  | -0.17 [-0.43, 0.08] | 0.180 |
| 6-<9 months before the RT-qPCR test | 26.23 (6.57) | -2.13 [-2.31, -1.94] | <0.001 |  | -0.66 [-0.85, -0.47] | <0.001 |
| ≥9 months before the RT-qPCR test | 26.07 (6.54) | -2.29 [-2.51, -2.07] | <0.001 |  | -0.67 [-0.90, -0.45] | <0.001 |
| Three doses |  |  |  |  |  |  |
| ≤1 month before the RT-qPCR test | 28.42 (6.59) | 0.06 [-0.29, 0.42] | 0.724 |  | 0.96 [0.62, 1.31] | <0.001 |
| >1 month before the RT-qPCR test | 27.29 (6.60) | -1.07 [-1.40, -0.74] | <0.001 |  | 0.29 [-0.03, 0.62] | 0.077 |
| **Previous SARS-CoV-2 infection** |  |  |  | <0.001 |  |  |
| Never | 26.97 (6.62) | Ref. |  |  | Ref. |  |
| <90 days before the study RT-qPCR test^**^ | 30.87 (4.92) | 3.90 [3.09, 4.71] | <0.001 |  | 4.67 [3.93, 5.42] | <0.001 |
| Prior infection^††^ | 28.24 (6.32) | 1.27 [1.02, 1.53] | <0.001 |  | 1.61 [1.37, 1.84] | <0.001 |

Abbreviations: CI, confidence interval; Ct, cycle threshold; RT-qPCR, real-time reverse-transcription polymerase chain reaction; Ref., reference; SARS-CoV-2, severe acute respiratory syndrome coronavirus 2; SD, standard deviation.

^*^The two-tailed F-test of the univariable analysis**.**

^†^RT-qPCR Ct value was adjusted for age-group, sex, nationality, Omicron subvariant, reason for RT-qPCR test, RT-qPCR test study-period week, vaccination status, and prior SARS-CoV-2 infection.

**^‡^**The 10-19 age group was chosen as a reference, and not the <10-age group, because of the different manifestations of this infection in small children.

^§^Nationalities were chosen to represent the most populous groups on Qatar.

^¶^These comprise 44 other nationalities in Qatar.

^**^An RT-qPCR-positive test that occurred <90 days before the study RT-qPCR-positive test was included separately in the analysis, but was not considered a prior infection. This RT-qPCR-positive test and the study RT-qPCR-positive test may both reflect the same prolonged infection.

^††^Prior infection was defined as an RT-qPCR-positive test that occurred ≥90 days before the RT-qPCR-positive test that is included in the study.

# **Table S3. Associations with RT-qPCR Ct value among 124,049 individuals with SARS-CoV-2 Omicron BA.2 subvariant infection.**

| **Characteristics** | **RT-qPCR Ct value** | **Univariable analysis** | | **F-test**^*^ | **Multivariable analysis^†^** | |
| --- | --- | --- | --- | --- | --- | --- |
|  | **Mean (SD)** | **β coefficient [95% CI]** | **p-value** | **p-value** | **β coefficient [95% CI]** | **p-value** |
| **Age group in years** |  |  |  | <0.001 |  |  |
| 10-19^‡^ | 23.73 (5.81) | Ref. |  |  | Ref. |  |
| <10 | 26.73 (5.72) | 3.00 [2.84, 3.16] | <0.001 |  | 2.95 [2.80, 3.11] | <0.001 |
| 20-29 | 23.50 (5.74) | -0.23 [-0.36, -0.10] | 0.001 |  | -0.09 [-0.22, 0.04] | 0.160 |
| 30-39 | 23.12 (5.68) | -0.61 [-0.74, -0.49] | <0.001 |  | -0.34 [-0.47, -0.22] | <0.001 |
| 40-49 | 23.12 (5.77) | -0.61 [-0.74, -0.47] | <0.001 |  | -0.40 [-0.54, -0.27] | <0.001 |
| 50-59 | 22.78 (5.80) | -0.95 [-1.11, -0.80] | <0.001 |  | -0.85 [-1.01, -0.70] | <0.001 |
| 60-69 | 22.79 (5.84) | -0.93 [-1.14, -0.73] | <0.001 |  | -1.07 [-1.27, -0.87] | <0.001 |
| 70-79 | 22.26 (5.70) | -1.47 [-1.83, -1.12] | <0.001 |  | -1.68 [-2.02, -1.34] | <0.001 |
| 80+ | 21.82 (5.51) | -1.90 [-2.44, -1.37] | <0.001 |  | -2.06 [-2.57, -1.55] | <0.001 |
| **Sex** |  |  |  | <0.001 |  |  |
| Female | 23.37 (5.85) | Ref. |  |  | Ref. |  |
| Male | 23.52 (5.80) | 0.16 [0.09, 0.22] | <0.001 |  | 0.20 [0.13, 0.26] | <0.001 |
| **Nationality**^§^ |  |  |  | <0.001 |  |  |
| Qatari | 23.81 (5.80) | Ref. |  |  | Ref. |  |
| Bangladeshi | 23.46 (6.04) | -0.36 [-0.55, -0.16] | <0.001 |  | 0.06 [-0.13, 0.25] | 0.512 |
| Egyptian | 22.75 (5.54) | -1.06 [-1.22, -0.90] | <0.001 |  | -0.45 [-0.60, -0.29] | <0.001 |
| Filipino | 22.35 (5.54) | -1.46 [-1.57, -1.34] | <0.001 |  | -0.96 [-1.08, -0.85] | <0.001 |
| Indian | 23.70 (5.96) | -0.11 [-0.21, -0.01] | 0.033 |  | -0.08 [-0.18, 0.03] | 0.150 |
| Nepalese | 24.44 (5.97) | 0.63 [0.47, 0.79] | <0.001 |  | 0.81 [0.65, 0.97] | <0.001 |
| Pakistani | 23.67 (5.95) | -0.15 [-0.34, 0.05] | 0.139 |  | 0.29 [0.11, 0.48] | 0.002 |
| Sri Lankan | 23.51 (5.83) | -0.30 [-0.51, -0.10] | 0.004 |  | -0.03 [-0.23, 0.16] | 0.731 |
| Sudanese | 23.43 (5.64) | -0.39 [-0.58, -0.20] | <0.001 |  | 0.57 [0.39, 0.75] | <0.001 |
| Other nationalities^¶^ | 23.44 (5.77) | -0.37 [-0.47, -0.27] | <0.001 |  | -0.12 [-0.21, -0.02] | 0.015 |
| **Reason for RT-qPCR testing** |  |  |  | <0.001 |  |  |
| Survey | 23.41 (5.84) | Ref. |  |  | Ref. |  |
| Clinical suspicion | 21.53 (5.20) | -1.88 [-2.00, -1.76] | <0.001 |  | -1.95 [-2.06, -1.84] | <0.001 |
| Contact tracing | 24.12 (5.91) | 0.71 [0.58, 0.84] | <0.001 |  | -0.50 [-0.63, -0.38] | <0.001 |
| Healthcare routine testing | 23.01 (5.66) | -0.40 [-0.67, -0.13] | 0.004 |  | -0.63 [-0.89, -0.37] | <0.001 |
| Port of entry | 25.72 (6.09) | 2.31 [2.11, 2.51] | <0.001 |  | 1.96 [1.77, 2.15] | <0.001 |
| Pre-travel | 24.58 (5.86) | 1.17 [1.06, 1.29] | <0.001 |  | 0.68 [0.57, 0.79] | <0.001 |
| Individual request | 23.60 (5.63) | 0.19 [0.03, 0.34] | 0.017 |  | -0.15 [-0.29, -0.00] | 0.048 |
| Other | 23.36 (5.34) | -0.05 [-0.72, 0.62] | 0.886 |  | -0.80 [-1.44, -0.16] | 0.014 |
| **RT-qPCR test study-period week** |  |  |  | <0.001 |  |  |
| Week 1 (23-29 December, 2021) | 22.02 (5.11) | Ref. |  |  | Ref. |  |
| Week 2 (30 December, 2021-05 January, 2022) | 22.60 (5.55) | 0.59 [0.46, 0.71] | <0.001 |  | 0.51 [0.39, 0.62] | <0.001 |
| Week 3 (06-12 January, 2022) | 23.52 (5.69) | 1.50 [1.38, 1.63] | <0.001 |  | 1.32 [1.19, 1.45] | <0.001 |
| Week 4 (13-19 January, 2022) | 25.04 (5.91) | 3.03 [2.87, 3.18] | <0.001 |  | 2.64 [2.49, 2.79] | <0.001 |
| Week 5 (20-26 January, 2022) | 26.99 (6.09) | 4.98 [4.79, 5.17] | <0.001 |  | 4.45 [4.27, 4.64] | <0.001 |
| Week 6 (27 January-02 February, 2022) | 27.53 (6.04) | 5.51 [5.28, 5.74] | <0.001 |  | 4.78 [4.56, 5.01] | <0.001 |
| Week 7 (03-09 February, 2022) | 28.07 (5.93) | 6.06 [5.77, 6.35] | <0.001 |  | 5.15 [4.87, 5.43] | <0.001 |
| Week 8 (10-16 February, 2022) | 27.37 (5.98) | 5.36 [4.97, 5.75] | <0.001 |  | 4.41 [4.03, 4.79] | <0.001 |
| Week 9 (17-20 February, 2022) | 27.33 (6.25) | 5.31 [4.67, 5.95] | <0.001 |  | 4.40 [3.78, 5.01] | <0.001 |
| **Vaccination status** |  |  |  | <0.001 |  |  |
| Unvaccinated | 24.55 (5.97) | Ref. |  |  | Ref. |  |
| One dose | 23.18 (5.66) | -1.36 [-1.75, -0.98] | <0.001 |  | -0.32 [-0.68, 0.04] | 0.083 |
| Two doses |  |  |  |  |  |  |
| <3 months before the RT-qPCR test | 23.89 (5.99) | -0.66 [-0.93, -0.39] | <0.001 |  | 0.31 [0.05, 0.56] | 0.017 |
| 3-<6 months before the RT-qPCR test | 23.39 (5.82) | -1.16 [-1.27, -1.04] | <0.001 |  | 0.01 [-0.10, 0.12] | 0.827 |
| 6-<9 months before the RT-qPCR test | 22.75 (5.59) | -1.80 [-1.88, -1.72] | <0.001 |  | -0.42 [-0.51, -0.33] | <0.001 |
| ≥9 months before the RT-qPCR test | 22.74 (5.59) | -1.81 [-1.91, -1.71] | <0.001 |  | -0.34 [-0.45, -0.24] | <0.001 |
| Three doses |  |  |  |  |  |  |
| ≤1 month before the RT-qPCR test | 24.19 (5.96) | -0.36 [-0.52, -0.21] | <0.001 |  | 0.84 [0.69, 0.99] | <0.001 |
| >1 month before the RT-qPCR test | 23.38 (5.86) | -1.17 [-1.32, -1.01] | <0.001 |  | 0.30 [0.15, 0.46] | <0.001 |
| **Previous SARS-CoV-2 infection** |  |  |  | <0.001 |  |  |
| Never | 23.35 (5.82) | Ref. |  |  | Ref. |  |
| <90 days before the study RT-qPCR test^**^ | 27.78 (5.40) | 4.42 [3.77, 5.07] | <0.001 |  | 3.95 [3.34, 4.56] | <0.001 |
| Prior infection^††^ | 24.43 (5.75) | 1.08 [0.96, 1.19] | <0.001 |  | 1.23 [1.12, 1.33] | <0.001 |

Abbreviations: CI, confidence interval; Ct, cycle threshold; RT-qPCR, real-time reverse-transcription polymerase chain reaction; Ref., reference; SARS-CoV-2, severe acute respiratory syndrome coronavirus 2; SD, standard deviation.

^*^The two-tailed F-test of the univariable analysis**.**

^†^RT-qPCR Ct value was adjusted for age-group, sex, nationality, Omicron subvariant, reason for RT-qPCR test, RT-qPCR test study-period week, vaccination status, and prior SARS-CoV-2 infection.

**^‡^**The 10-19 age group was chosen as a reference, and not the <10-age group, because of the different manifestations of this infection in small children.

^§^Nationalities were chosen to represent the most populous groups on Qatar.

^¶^These comprise 44 other nationalities in Qatar.

^**^An RT-qPCR-positive test that occurred <90 days before the study RT-qPCR-positive test was included separately in the analysis, but was not considered a prior infection. This RT-qPCR-positive test and the study RT-qPCR-positive test may both reflect the same prolonged infection.

^††^Prior infection was defined as an RT-qPCR-positive test that occurred ≥90 days before the RT-qPCR-positive test that is included in the study.

**Table S4. STROBE checklist for cross-sectional studies.**

|  | **Item No** | **Recommendations** | **Main text page No** |
| --- | --- | --- | --- |
| **Title and abstract** | 1 | (*a*) Indicate the study’s design with a commonly used term in the title or the abstract | Section S2 (‘Study population, data sources, and study design’, paragraph 1) of Supplementary Appendix |
|  |  | (*b*) Provide in the abstract an informative and balanced summary of what was done and what was found | NA |
| **Introduction** | | |  |
| Background/rationale | 2 | Explain the scientific background and rationale for the investigation being reported | Main text p.3 |
| Objectives | 3 | State specific objectives, including any prespecified hypotheses | Main text p.3, & Section S2 (‘Study population, data sources, and study design’) of Supplementary Appendix |
| **Methods** | | |  |
| Study design | 4 | Present key elements of study design early in the paper | Main text p.3, & Section S2 (‘Study population, data sources, and study design’) of Supplementary Appendix |
| Setting | 5 | Describe the setting, locations, and relevant dates, including periods of recruitment, exposure, follow-up, and data collection | Main text p.3, & Sections S1 (‘Classification of infections by variant type’) & S2 (‘Study population, data sources, and study design’) of Supplementary Appendix |
| Participants | 6 | (*a*) Give the eligibility criteria, and the sources and methods of selection of participants | Main text p.3 & p.4, & Section S2 (‘Study population, data sources, and study design’) & Figure S2 of Supplementary Appendix |
| Variables | 7 | Clearly define all outcomes, exposures, predictors, potential confounders, and effect modifiers. Give diagnostic criteria, if applicable | Main text p.3, & Sections S1 (‘Classification of infections by variant type’) & S2 (‘Study population, data sources, and study design’ & ‘Statistical analysis’) of Supplementary Appendix |
| Data sources/ measurement | 8 | For each variable of interest, give sources of data and details of methods of assessment (measurement). Describe comparability of assessment methods if there is more than one group | Main text p.3, Sections S1 & S2 (‘Study population, data sources, and study design’), & Table S1 of Supplementary Appendix |
| Bias | 9 | Describe any efforts to address potential sources of bias | Main text p.3, & Section S2 (‘Study population, data sources, and study design’ & ‘Statistical analysis’) of Supplementary Appendix |
| Study size | 10 | Explain how the study size was arrived at | Section S2 (‘Study population, data sources, and study design’) & Figure S2 of Supplementary Appendix |
| Quantitative variables | 11 | Explain how quantitative variables were handled in the analyses. If applicable, describe which groupings were chosen and why | Table 1, & Section S2 (‘Study population, data sources, and study design’ & ‘Statistical analysis’), & Tables S1, S2, & S3 of Supplementary Appendix |
| Statistical methods | 12 | (*a*) Describe all statistical methods, including those used to control for confounding | Section S2 (‘Statistical analysis’) of Supplementary Appendix |
|  |  | (*b*) Describe any methods used to examine subgroups and interactions | Main text p.4, & Section S2 (‘Statistical analysis’) & Tables S1, S2 & S3 of Supplementary Appendix |
|  |  | (*c*) Explain how missing data were addressed | Section S2 (‘Study population, data sources, and study design’) & Figure S2 of Supplementary Appendix |
|  |  | (*d*) If applicable, describe analytical methods taking account of sampling strategy | NA |
|  |  | (*e*) Describe any sensitivity analyses | NA |
| **Results** | | |  |
| Participants | 13 | (a) Report numbers of individuals at each stage of study—eg numbers potentially eligible, examined for eligibility, confirmed eligible, included in the study, completing follow-up, and analysed | Main text p.3, & Figure S2 of Supplementary Appendix |
|  |  | (b) Give reasons for non-participation at each stage |  |
|  |  | (c) Consider use of a flow diagram |  |
| Descriptive data | 14 | (a) Give characteristics of study participants (eg demographic, clinical, social) and information on exposures and potential confounders | Main text p.3, & Table S1 of Supplementary Appendix |
|  |  | (b) Indicate number of participants with missing data for each variable of interest | Section S2 (‘Study population, data sources, and study design’) & Figure S2 of Supplementary Appendix |
| Outcome data | 15 | Report numbers of outcome events or summary measures | Table 1 |
| Main results | 16 | (*a*) Give unadjusted estimates and, if applicable, confounder-adjusted estimates and their precision (eg, 95% confidence interval). Make clear which confounders were adjusted for and why they were included | Main text p.4 & Table 1 |
|  |  | (*b*) Report category boundaries when continuous variables were categorized | Table 1, & Tables S1, S2, & S3 of Supplementary Appendix |
|  |  | (*c*) If relevant, consider translating estimates of relative risk into absolute risk for a meaningful time period | NA |
| Other analyses | 17 | Report other analyses done—eg analyses of subgroups and interactions, and sensitivity analyses | Main text p.4, & Tables S2 & S3 of Supplementary Appendix |
| **Discussion** | | |  |
| Key results | 18 | Summarise key results with reference to study objectives | Main text p.4 |
| Limitations | 19 | Discuss limitations of the study, taking into account sources of potential bias or imprecision. Discuss both direction and magnitude of any potential bias | Section S2 (‘Limitations’) of Supplementary Appendix |
| Interpretation | 20 | Give a cautious overall interpretation of results considering objectives, limitations, multiplicity of analyses, results from similar studies, and other relevant evidence | Main text p.4, & Section S2 (‘Limitations’) of Supplementary Appendix |
| Generalisability | 21 | Discuss the generalisability (external validity) of the study results | Section S2 (‘Limitations’) |
| **Other information** | | |  |
| Funding | 22 | Give the source of funding and the role of the funders for the present study and, if applicable, for the original study on which the present article is based | Main text p.8 |

Abbreviations: NA: not applicable; not applicable; p. page; Supp. Supplementary Appendix.

# **Supplementary references**

1 C Vogels, J Fauver, N Grubaugh. Multiplexed rt-qpcr to screen for sars-cov-2 b.1.1.7, b.1.351, and p.1 variants of concern v.3. Dx.Doi.Org/10.17504/protocols.Io.Br9vm966. ed.^,eds., 2021.

2 LJ Abu-Raddad, H Chemaitelly, AA Butt, National Study Group for Covid Vaccination. Effectiveness of the bnt162b2 covid-19 vaccine against the b.1.1.7 and b.1.351 variants. N Engl J Med 2021; 385(2):187-189.

3 H Chemaitelly, HM Yassine, FM Benslimane, et al. Mrna-1273 covid-19 vaccine effectiveness against the b.1.1.7 and b.1.351 variants and severe covid-19 disease in qatar. Nat Med 2021; 27(9):1614-1621.

4 National Project of Surveillance for Variants of Concern and Viral Genome Sequencing. Qatar viral genome sequencing data. Data on randomly collected samples. <Https://www.Gisaid.Org/phylodynamics/global/nextstrain/>. ed.^,eds., 2021.

5 FM Benslimane, HA Al Khatib, O Al-Jamal, et al. One year of sars-cov-2: Genomic characterization of covid-19 outbreak in qatar. Front Cell Infect Microbiol 2021; 11(768883.

6 MR Hasan, MKR Kalikiri, F Mirza, et al. Real-time sars-cov-2 genotyping by high-throughput multiplex pcr reveals the epidemiology of the variants of concern in qatar. Int J Infect Dis 2021; 112(52-54.

7 H Chemaitelly, P Tang, MR Hasan, et al. Waning of bnt162b2 vaccine protection against sars-cov-2 infection in qatar. N Engl J Med 2021; 385(24):e83.

8 J Saththasivam, SS El-Malah, TA Gomez, et al. Covid-19 (sars-cov-2) outbreak monitoring using wastewater-based epidemiology in qatar. Sci Total Environ 2021; 774(145608.

9 World Health Organization. Tracking sars-cov-2 variants. Available from: <Https://www.Who.Int/en/activities/tracking-sars-cov-2-variants/>. 2021.

10 LJ Abu-Raddad, H Chemaitelly, HH Ayoub, et al. Effectiveness of bnt162b2 and mrna-1273 covid-19 boosters against sars-cov-2 omicron (b.1.1.529) infection in qatar. medRxiv In press at New England Journal of Medicine 2022:2022.01.18.22269452.

11 H Chemaitelly, HH Ayoub, P Coyle, et al. Protection of omicron sub-lineage infection against reinfection with another omicron sub-lineage. medRxiv 2022:2022.02.24.22271440.

12 HN Altarawneh, H Chemaitelly, MR Hasan, et al. Protection against the omicron variant from previous sars-cov-2 infection. N Engl J Med 2022.

13 H Chemaitelly, HH Ayoub, S AlMukdad, et al. Duration of protection of bnt162b2 and mrna-1273 covid-19 vaccines against symptomatic sars-cov-2 omicron infection in qatar. medRxiv 2022:2022.02.07.22270568.

14 Thermo Fisher Scientific. Taqpath™ covid‑19 ce‑ivd rt‑pcr kit instructions for use. Available from: <Https://assets.Thermofisher.Com/tfs-assets/lsg/manuals/man0019215_taqpathcovid-19_ce-ivd_rt-pcr%20kit_ifu.Pdf>. Accessed on december 02, 2020. 2020.

15 UK Health Security Agency. Sars-cov-2 variants of concern and variants under investigation in england: Technical briefing 34. ed.^,eds. England, 2022.

16 A Singanayagam, M Patel, A Charlett, et al. Duration of infectiousness and correlation with rt-pcr cycle threshold values in cases of covid-19, england, january to may 2020. Euro Surveill 2020; 25(32).

17 FP Lyngse, K Mølbak, K Træholt Franck, et al. Association between sars-cov-2 transmissibility, viral load, and age in households. medRxiv 2021:2021.02.28.21252608.

18 LYW Lee, S Rozmanowski, M Pang, et al. Sars-cov-2 infectivity by viral load, s gene variants and demographic factors and the utility of lateral flow devices to prevent transmission. Clin Infect Dis 2021.

19 M Marks, P Millat-Martinez, D Ouchi, et al. Transmission of covid-19 in 282 clusters in catalonia, spain: A cohort study. Lancet Infect Dis 2021; 21(5):629-636.

20 J Bullard, K Dust, D Funk, et al. Predicting infectious severe acute respiratory syndrome coronavirus 2 from diagnostic samples. Clin Infect Dis 2020; 71(10):2663-2666.

21 LJ Abu-Raddad, H Chemaitelly, HH Ayoub, et al. Relative infectiousness of sars-cov-2 vaccine breakthrough infections, reinfections, and primary infections. Nat Commun 2022; 13(1):532.

22 FP Polack, SJ Thomas, N Kitchin, et al. Safety and efficacy of the bnt162b2 mrna covid-19 vaccine. N Engl J Med 2020; 383(27):2603-2615.

23 LR Baden, HM El Sahly, B Essink, et al. Efficacy and safety of the mrna-1273 sars-cov-2 vaccine. N Engl J Med 2021; 384(5):403-416.

24 LJ Abu-Raddad, H Chemaitelly, HH Ayoub, et al. Characterizing the qatar advanced-phase sars-cov-2 epidemic. Sci Rep 2021; 11(1):6233.

25 P Tang, MR Hasan, H Chemaitelly, et al. Bnt162b2 and mrna-1273 covid-19 vaccine effectiveness against the sars-cov-2 delta variant in qatar. Nat Med 2021; 27(12):2136-2143.

26 H Chemaitelly, R Bertollini, LJ Abu-Raddad, National Study Group for Covid Epidemiology. Efficacy of natural immunity against sars-cov-2 reinfection with the beta variant. N Engl J Med 2021; 385(27):2585-2586.

27 Planning and Statistics Authority-State of Qatar. Qatar monthly statistics. Available from: <Https://www.Psa.Gov.Qa/en/pages/default.Aspx>. Accessed on: May 26, 2020. 2020.

28 LJ Abu-Raddad, H Chemaitelly, HH Ayoub, et al. Association of prior sars-cov-2 infection with risk of breakthrough infection following mrna vaccination in qatar. JAMA 2021; 326(19):1930-1939.

29 LJ Abu-Raddad, H Chemaitelly, P Coyle, et al. Sars-cov-2 antibody-positivity protects against reinfection for at least seven months with 95% efficacy. EClinicalMedicine 2021; 35(100861.

30 LJ Abu-Raddad, H Chemaitelly, JA Malek, et al. Assessment of the risk of severe acute respiratory syndrome coronavirus 2 (sars-cov-2) reinfection in an intense reexposure setting. Clin Infect Dis 2021; 73(7):e1830-e1840.

31 CH Hansen, D Michlmayr, SM Gubbels, et al. Assessment of protection against reinfection with sars-cov-2 among 4 million pcr-tested individuals in denmark in 2020: A population-level observational study. Lancet 2021; 397(10280):1204-1212.

32 N Kojima, NK Shrestha, JD Klausner. A systematic review of the protective effect of prior sars-cov-2 infection on repeat infection. Eval Health Prof 2021; 44(4):327-332.

33 S Pilz, V Theiler-Schwetz, C Trummer, et al. Sars-cov-2 reinfections: Overview of efficacy and duration of natural and hybrid immunity. Environ Res 2022:112911.

34 LJ Abu-Raddad, H Chemaitelly, R Bertollini, National Study Group for Covid Epidemiology. Severity of sars-cov-2 reinfections as compared with primary infections. N Engl J Med 2021; 385(26):2487-2489.

35 LJ Abu-Raddad, H Chemaitelly, HH Ayoub, et al. Introduction and expansion of the sars-cov-2 b.1.1.7 variant and reinfections in qatar: A nationally representative cohort study. PLoS Med 2021; 18(12):e1003879.

36 HH Ayoub, M Tomy, H Chemaitelly, et al. Estimating protection afforded by prior infection in preventing reinfection: Applying the test-negative study design. medRxiv 2022:2022.01.02.22268622.

37 V Nussenblatt, AE Roder, S Das, et al. Year-long covid-19 infection reveals within-host evolution of sars-cov-2 in a patient with b cell depletion. medRxiv 2021.

38 B Choi, MC Choudhary, J Regan, et al. Persistence and evolution of sars-cov-2 in an immunocompromised host. N Engl J Med 2020; 383(23):2291-2293.

39 LJ Abu-Raddad, H Chemaitelly, JA Malek, et al. Two prolonged viremic sars-cov-2 infections with conserved viral genome for two months. Infect Genet Evol 2021; 88(104684.

40 StataCorp. Statistical software: Release 16.1. College Station, TX: Stata Corporation 2019.

41 S Seedat, H Chemaitelly, HH Ayoub, et al. Sars-cov-2 infection hospitalization, severity, criticality, and fatality rates in qatar. Sci Rep 2021; 11(1):18182.

42 HH Ayoub, H Chemaitelly, S Seedat, et al. Mathematical modeling of the sars-cov-2 epidemic in qatar and its impact on the national response to covid-19. J Glob Health 2021; 11(05005.

43 PV Coyle, H Chemaitelly, MA Ben Hadj Kacem, et al. Sars-cov-2 seroprevalence in the urban population of qatar: An analysis of antibody testing on a sample of 112,941 individuals. iScience 2021; 24(6):102646.

44 MH Al-Thani, E Farag, R Bertollini, et al. Sars-cov-2 infection is at herd immunity in the majority segment of the population of qatar. Open Forum Infect Dis 2021; 8(8):ofab221.

45 A Jeremijenko, H Chemaitelly, HH Ayoub, et al. Herd immunity against severe acute respiratory syndrome coronavirus 2 infection in 10 communities, qatar. Emerg Infect Dis 2021; 27(5):1343-1352.

46 R Challen, E Brooks-Pollock, JM Read, et al. Risk of mortality in patients infected with sars-cov-2 variant of concern 202012/1: Matched cohort study. BMJ 2021; 372(n579.
